# At Term, XmO and XpO Mouse Placentas Show Differences in Glucose Metabolism in the Trophectoderm-Derived Outer Zone

**DOI:** 10.3389/fcell.2017.00063

**Published:** 2017-06-21

**Authors:** Nannan He, Shujing J. Lim, Joana C. Moreira de Mello, Injerreau Navarro, Monika Bialecka, Daniela C. F. Salvatori, Lucette A. J. van der Westerlaken, Lygia V. Pereira, Susana M. Chuva de Sousa Lopes

**Affiliations:** ^1^Department of Anatomy and Embryology, Leiden University Medical CenterLeiden, Netherlands; ^2^Department of Genetics and Evolutionary Biology, University of São PauloSão Paulo, Brazil; ^3^Central Laboratory Animal Facility, Leiden University Medical CenterLeiden, Netherlands; ^4^Department of Gynaecology, Leiden University Medical CenterLeiden, Netherlands; ^5^Department for Reproductive Medicine, Ghent University HospitalGhent, Belgium

**Keywords:** X chromosome, mouse, placenta, trophoblast cells, glucose metabolism, Turner Syndrome

## Abstract

Genetic mouse model (39,XO) for human Turner Syndrome (45,XO) harboring either a single maternally inherited (Xm) or paternally inherited (Xp) chromosome show a pronounced difference in survival rate at term. However, a detailed comparison of XmO and XpO placentas to explain this difference is lacking. We aimed to investigate the morphological and molecular differences between XmO and XpO term mouse placentas. We observed that XpO placentas at term contained a significantly larger area of glycogen cells (GCs) in their outer zone, compared to XmO, XX, and XY placentas. In addition, the outer zone of XpO placentas showed higher expression levels of lactate dehydrogenase (*Ldha*) than XmO, XX, and XY placentas, suggestive of increased anaerobic glycolysis. In the labyrinth, we detected significantly lower expression level of trophectoderm (TE)-marker keratin 19 (*Krt19*) in XpO placentas than in XX placentas. The expression of other TE-markers was comparable as well as the area of TE-derived cells between XO and wild-type labyrinths. XpO placentas exhibited specific defects in the amount of GCs and glucose metabolism in the outer zone, suggestive of increased anaerobic glycolysis, as a consequence of having inherited a single Xp chromosome. In conclusion, the XpO genotype results in a more severe placental phenotype at term, with distinct abnormalities regarding glucose metabolism in the outer zone.

## Introduction

Turner Syndrome is the most common sex chromosome disorder affecting 1 in 2,000 live births. It is caused by the loss of genetic material from one of the sex chromosomes and the retained single X chromosome can be inherited either from the mother (Xm) or the father (Xp) (Saenger, 1996). In humans, 99% of the XO fetuses are lost during pregnancy (Cockwell et al., 1991; Ranke and Saenger, 2001). By contrast, in mice, 90% of embryos with a single Xm survive to term, whereas 40% of embryos with a single Xp are resorbed due to severe placental abnormalities (Burgoyne et al., 1983a,b; Hunt, 1991). This suggests that the transcription of a single Xm or Xp in mouse placentas is not equivalent and influences development differently, reflecting either a different genome-wide epigenetic landscape between Xp and Xm or the existence of certain paternally imprinted X-linked genes in the mouse placenta.

X chromosome inactivation (XCI) is better understood in mice than in humans (Payer and Lee, 2008, 2014; Okamoto et al., 2011; Deng et al., 2014; Petropoulos et al., 2016) due to the existence of well-studied genetic sub-strains of mice. In mice, in female (XX) late blastocysts, the trophectoderm (TE), and primitive endoderm (PE) show imprinted XCI (with an obligatory active Xm) and the epiblast (EPI) shows random XCI (in each cell either the Xm or Xp is active; Payer and Lee, 2008; Silva et al., 2009; Okamoto et al., 2011). In the placenta, the TE-derived cells [trophoblast giant cells (TGCs), spongiotrophoblasts and glycogen cells (GCs) in the outer zone; mononuclear trophoblast cells and syncytiotrophoblast cells in the labyrinth] maintain imprinted XCI (active Xm). By contrast, the chorionic plate and the embryonic endothelial cells of the labyrinth are derived from EPI, therefore showing random XCI.

In humans, there may not be imprinted XCI in the placenta (de Mello et al., 2010; Penaherrera et al., 2012; Hamada et al., 2016). By the end of the first trimester, the placental volume of XO and control placenta seemed comparable (Wegrzyn et al., 2005); and the birth weight of XmO and XpO new-born babies was similar (Mathur et al., 1991). Nevertheless, there is a higher incidence of XpO human fetuses lost during pregnancy (Jacobs et al., 1989) and the percentage of patients retaining the XmO is 60%–80% (Monroy et al., 2002; Uematsu et al., 2002; Sagi et al., 2007; Ko et al., 2010; Álvarez-Nava et al., 2013).

The placenta is a crucial organ during mammalian development, ensuring the selective and directional transport of gases, nutrients and waste products between the maternal blood and the embryonic blood (Jansson, 2016). In mice, the GCs may serve as a potential additional energy source, due to their high glycogen content and sensitivity to glucagon signaling (Coan et al., 2006). The placenta is a highly regulative organ that adapts constantly to the maternal environment, for example oxygen tension and hypoxia (Adelman et al., 2000; Higgins et al., 2016), availability of nutrients or calorie restriction (Ganguly et al., 2012) and exposure to maternal hormones (Fowden et al., 2009; Dimasuay et al., 2016) to sustain optimal embryonic growth throughout pregnancy.

Interestingly, (epi)genetic abnormalities that affect placental development trigger an adaptive response in the placenta to suppress the decreased efficiency to support embryonic growth (Hemberger, 2002; Lefebvre, 2012; Sandovici et al., 2012; Himes et al., 2013). In the case of XO embryos, embryonic day (E)8.5 XpO embryos had been shown to have small ectoplacental cones (Jamieson et al., 1998). However, by E14, XpO placentas had caught up in size and some showed a larger outer zone (Zechner et al., 1997). At E18.5, XpO placentas were significantly heavier than XX controls (Burgoyne et al., 1983b). But in a later study, XmO, XpO, and XY placentas were found heavier than XX placentas (Ishikawa et al., 2003).

To date, a detailed comparison of XmO and XpO term placentas, in particular the TE-derived part of the placenta, is missing. Here, we show that E18.5 XpO placentas exhibited significantly larger area occupied by GCs in outer zone when compared to XmO, XX, and XY placentas. Moreover, the expression of *Ldha*, coding for the enzyme that converts lactate to pyruvate through anaerobic glycolysis, was significantly higher in outer zone of XpO placentas than the XmO, XX, and XY placentas, suggesting increased anaerobic glycolysis and underlying possible defects in oxygen availability in XpO placentas. In conclusion, the XpO genotype results in a more severe placental phenotype at term, with distinct abnormalities regarding glucose metabolism in the outer zone.

## Materials and methods

### Mice and genotyping

All animal tissues used in this work were a generous gift from P. Burgoyne in accordance with the United Kingdom Animals Scientific Procedures Act 1986 and approved by the local ethical committee of the National Institute of Medical Research, London. MF1 mice bearing XX and XY embryos were from XX × XY crossings. XmO and XpO mice were generated as previously described (Ishikawa et al., 2003). Briefly, XmO animals were produced by crossing XX females with X^Y^O males, and identified by visual detection of female genitalia; the X^Y^O males were generated by crossing X^Paf^O females with XY^*^ males. The XpO animals were generated by crossing In(X)^Paf^/X females with XY males. All females [In(X)/X, XX^Paf^, and XpO] were karyotyped with trypsin-Giemsa banding using fresh liver to identify the XpO embryos (with 39 chromosomes as opposed to 40 chromosomes).

### Placenta collection and histology

From a total of 12 litters, E18.5 embryos were isolated in phosphate buffer saline (PBS) and separated into males and females by morphology and genotyped as above. The placentas were dissected into quarters and some quarters were collected for RNA isolation after removal of the outer zone (Jz, TGCs, and decidua), whereas others were fixed in 4% paraformaldehyde (PFA, Merck, Darmstadt, Germany) at 4°C overnight (o/n), washed in PBS and dehydrated through increasing concentrations of ethanol and finally xylene, embedded in paraffin and serially sectioned (5 μm) in the sagittal plane using a microtome (Leica RM2055, Nussloch, Germany) in the medial-to-lateral direction (*N* = 3 XX, *N* = 3 XY, *N* = 5 XmO, *N* = 4 XpO).

Prior to Periodic acid-Shiff (PAS) staining, sections were deparaffinised in xylene, rehydrated through a series of ethanol solutions and incubated 30 min at 56°C in pre-heated 1% periodic acid (Sigma-Aldrich, St. Louis, USA), rinsed in water, immersed in Schiff's reagent (Klinipath, Duiven, The Netherlands) 30 min at room temperature (RT), rinsed in water and counterstained with Mayer's haematoxylin (Merck, Darmstadt, Germany). Congo red, Masson's trichrome and Hematoxylin-eosin staining were performed using standard histological procedures. Stained sections were washed in water, dehydrated through a series of ethanol, xylene, and mounted in Entellan (Merck, Darmstadt, Germany).

### Quantitative reverse-transcription polymerase chain reaction (QPCR)

QPCR was performed on placental quarters after removal of the outer zone and analyzed as described (de Melo Bernardo et al., 2015); or on RNA material isolated from 5x paraffin sections of the outer zone using RecoverAll total nucleic acid isolation kit (AM1975, Ambion, Carlsbad, CA, USA) following the manufacturer's protocol. For normalization, the ΔΔCt method was used with the reference genes *Ubc* and *B2m*, stably expressed in mouse placenta (Solano et al., 2016). All individual placentas were analyzed in technical triplicates. The fold change in expression was calculated relative to the XX1 placenta. Briefly, the average ΔCt value from the technical triplicates of the XX1 (Ave ΔCt XX1) was calculated. Next, we subtracted Ave ΔCt XX1 from each ΔCt (ΔΔCt) and the relative fold change [2^−(ΔΔCt)^] calculated. The fold change of each of the triplicate values per placenta was then averaged (mean) and the standard deviation was calculated. The primers used are listed in Supplementary Table 1.

### Immunofluorescence

Paraffin sections were deparaffinised and used for immunofluorescence as previously described (Heeren et al., 2015). Primary antibodies used were rabbit anti-KRT19 (or keratin 19) (1:250; ab52625, Abcam, Cambridge, UK) and rat anti-EMCN (or endomucin) (1:150; sc-65495, Santa Cruz Biotechnology, Santa Cruz, CA, USA). Afterwards, sections were washed in 0.05% Tween-20/PBS, treated with 0.3% Sudan Black B (Edward Gurr Ltd, London, UK) in 70% ethanol for 5 min to eliminate background autofluorescence from red blood cells (Romijn et al., 1999) and incubated with secondary antibodies diluted in blocking solution for 1 h at RT. Secondary antibodies were Alexa Fluor 488 goat anti-rabbit (1:500; A-11034, Life Technologies, Eugene, OR, USA) and Alexa Fluor 555 goat anti-rat (1:500; A-21434, Life Technologies, Eugene, OR, USA). Nuclei were stained with 4′,6-diamidino-2-phenylindole (DAPI) (Vector Laboratories, Peterborough, UK) and sections were mounted in Prolong Gold anti-fade reagent (Life technologies, Eugene, OR, USA). Slides used for isotype controls were treated as above using rabbit immunoglobulin fraction (1:250; X0903, Dako, Heverlee, Belgium) and rat IgG2a (1:150, MAB006, R&D Systems, Minneapolis, MN, USA) instead of the primary antibodies.

### Imaging and quantification

Bright-field images were taken on an Olympus AX70 microscope (Olympus, Zoeterwoude, Netherlands) equipped with a digital camera (Olympus XC50, Tokyo, Japan). Fluorescence images were acquired on a Leica DMRA fluorescence microscope (Leica, Wetzlar, Germany) with a CoolSnap HQ2 camera (Photometrics, Tucson, USA) or a Leica AF6000 fluorescence microscope with a Hamamatsu EM-CCD C9100 camera (Leica Microsystems, Wetzlar, Germany). Quantification was performed in ImageJ 1.48 (http://imagej.nih.gov/ij).

### Statistical analysis

The statistical analyses of the proportion of glycogen cells area in the outer zone of the placentas, the percentage of fetal vascular space, the area occupied by TE-derived cells in the labyrinth and differential gene expression per genotype were performed using one-way ANOVA with the Tukey-HSD applied for *post-hoc* testing, using statistical software package SPSS 20.0 (SPSS Inc., Chicago, IL, USA). *P* < 0.05 was considered significant.

## Results

### At term, XpO placentas showed larger area occupied by GCs in the outer zone

**Figure 1:**
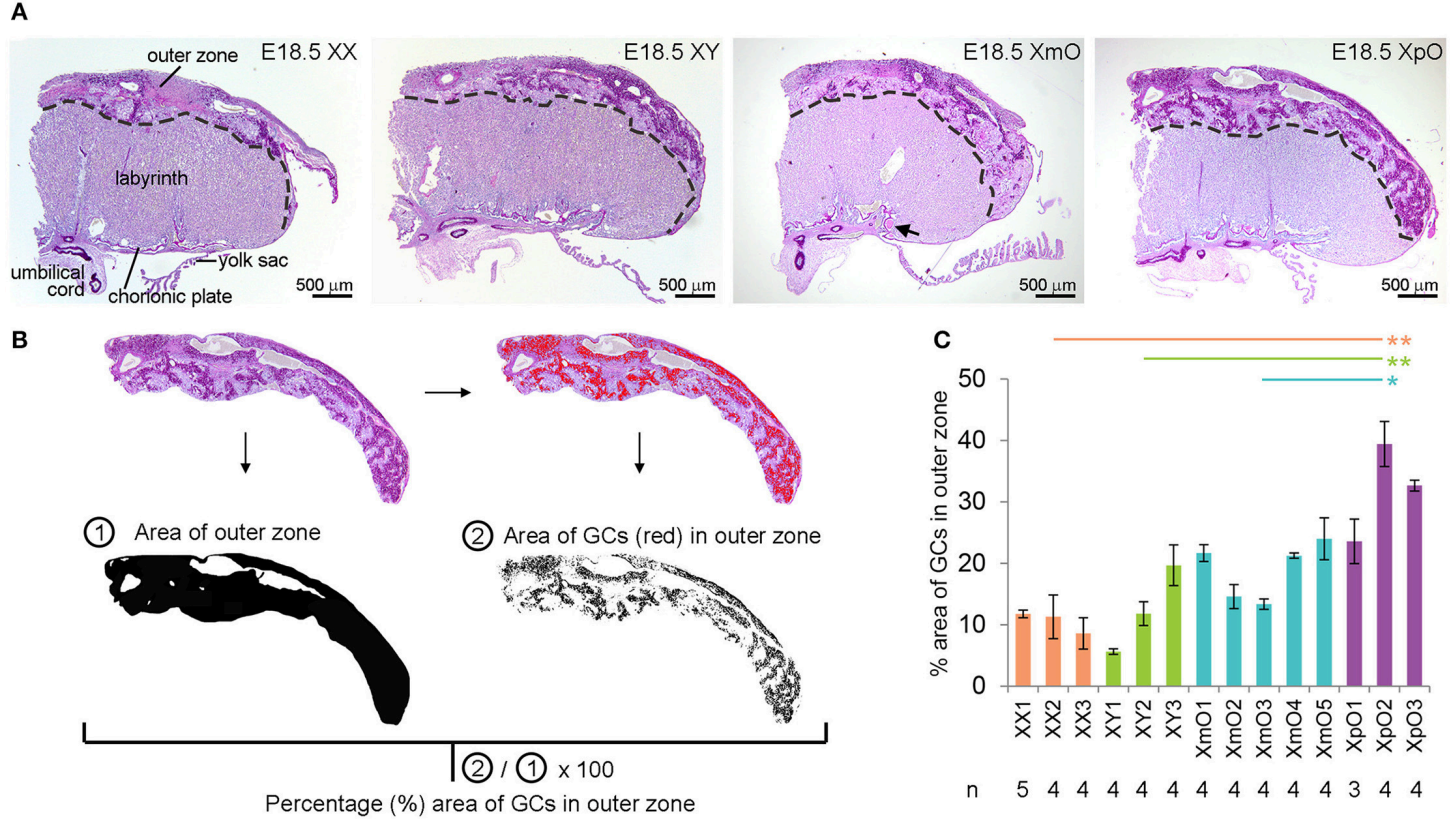
XpO placentas have a larger area occupied by glycogen cells in the outer zone. **(A)** Representative PAS-stained medial placental sections of the different genotypes (XX, XY, XmO, XpO). **(B)** From the images acquired by microscopy, the outer zone of the placenta is digitally selected, then converted to black-and-white on Image J to provide the total area of the outer zone. From the selected outer zone of the placenta, the dark PAS-stained glycogen cells (GCs) are identified digitally (red cells) by selecting a threshold on Image J. The percentage of the GCs in the outer zone of each placenta section is obtained by using the ratio of the area of GCs ②/total area of the outer zone ①. **(C)** Graph depicting the percentage (%) area of outer zone occupied by GCs in the different genotypes (XX, XY, XmO, XpO). Significant *P*-values between XpO placentas and the other genotypes are indicated by ^*^P < 0.05 and ^**^P < 0.01.

We investigated the placental morphology of the four genotypes (*N* = 3 XX, *N* = 3 XY, *N* = 5 XmO, *N* = 3 XpO) at E18.5 using PAS-staining. This allowed us to distinguish the outer zone from the labyrinth (Figure 1A). We observed the presence of a broader outer zone in the lateral part of XmO (*N* = 2 in 5, 40%) and XpO (*N* = 3 in 3, 100%) placentas (Figure 1A). To quantify the area occupied by GCs in the outer zone, the ratio of the area occupied by GCs in the outer zone was calculated on individual placental sections (*n* = 3–5 medial sections per placenta, containing a visible connection to the umbilical cord; Figures 1B,C). The XpO placentas contained a significantly larger area occupied by GCs in the outer zone when compared to XX, XY, and XmO placentas (*P* = 0.004, 0.008, and 0.045, respectively, Figure 1C). In contrast, the area occupied by GCs in the outer zone of XmO placentas was comparable to that of XX and XY placentas (Figure 1C).

### At term, XpO placentas showed increased *Ldha* expression in the outer zone

**Figure 2:**
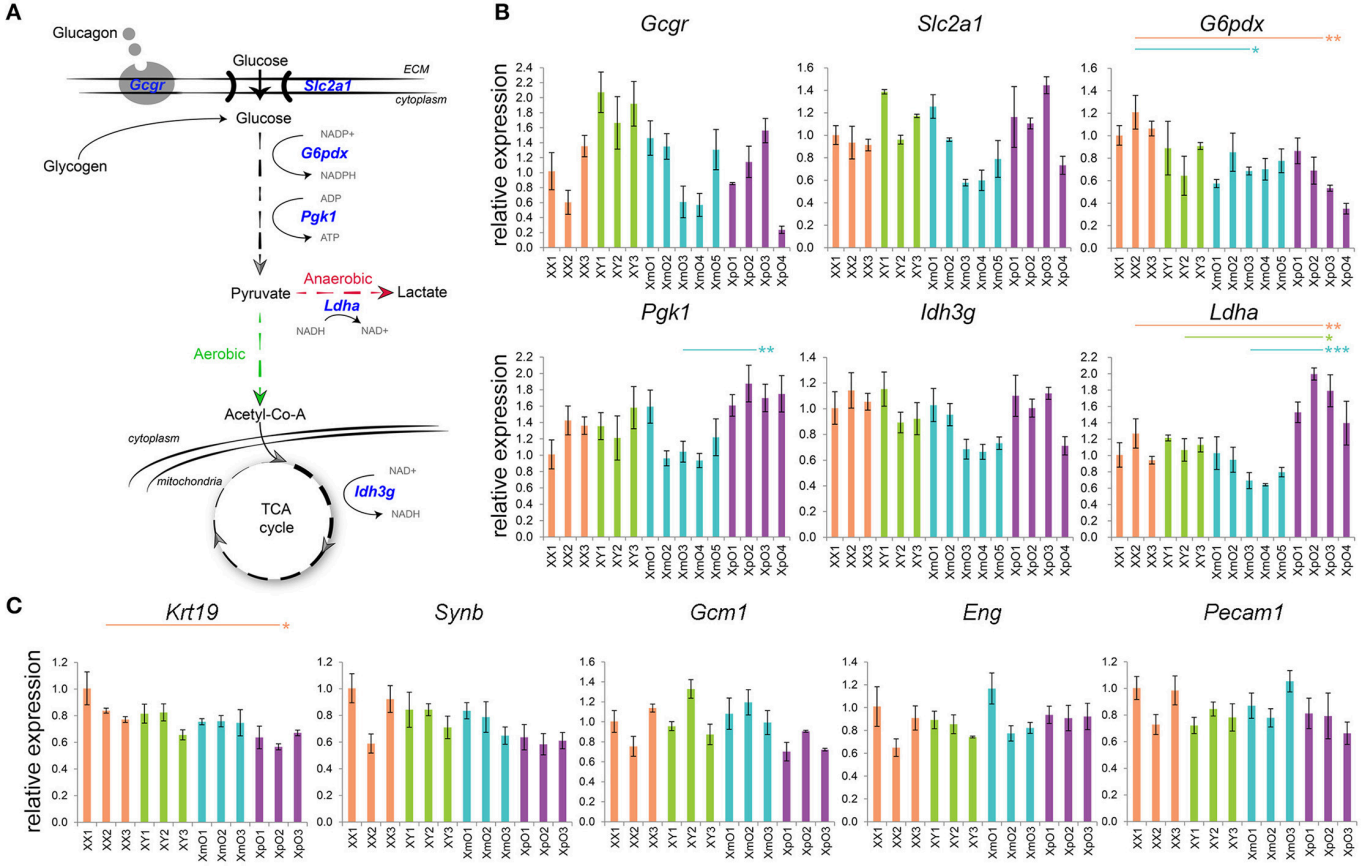
Expression of genes related to glucose metabolism in the outer zone and expression of genes related to TE and vasculature in the labyrinth. **(A)** Scheme of glucose metabolism cascade, with the genes analyzed in blue. **(B)** Relative expression of the depicted genes in the outer zone of XX, XY, XmO, XpO placentas. Each bar represents mean ± standard deviation of technical triplicates of a different individual placenta. *P*-values were calculated between the different genotype-groups using one-way ANOVA with the Tukey-HSD. Significant *P*-values between genotypes are indicated by ^*^P < 0.05, ^**^P < 0.01, and ^***^P < 0.001. **(C)** Relative expression of the depicted genes in the labyrinth of XX, XY, XmO, XpO placentas. Each bar represents mean ± standard deviation of technical triplicates of a different individual placenta. Significant *P*-value between XX and XpO is indicated by ^*^P < 0.05.

The larger area of GCs in outer zone of XpO placentas led us to investigate defects in gene expression related to glucagon signaling and glucose metabolism (Figure 2A) in the outer zone. The expression level of glucagon receptor (*Gcgr*) as well as of glucose transporter *Slc2a1*, which mediate passive glucose uptake in cells (Zhao and Keating, 2007) was similar between XmO, XpO, and XX placentas (Figure 2B, Supplementary Table 2).

Two X-linked genes, *G6pdx* and *Pgk1*, encode essential enzymes in the conversion of glucose to pyruvate (Semenza et al., 1994; Tuttle et al., 2000). In the outer zone, *G6pdx* showed significantly lower expression in both XmO and XpO placentas compared to the XX placentas (XmO vs. XX: *P* = 0.028; XpO vs. XX: *P* = 0.007; Figure 2B, Supplementary Table 2). However, expression of *Pgk1* was significantly higher in XpO placentas than in XmO placentas (*P* = 0.009, Figure 2B, Supplementary Table 2).

Under normal oxygen supply (aerobic glycolysis), pyruvate is catabolized into acetyl-CoA to be used in the tricarboxylic acid (TCA) cycle to produce energy efficiently (Figure 2A). One of the enzymes of the TCA cycle, encoded by X-linked gene *Idh3g*, showed similar expression between XO and wild-type placentas (Figure 2B, Supplementary Table 2). However, if oxygen supply is low, pyruvate is metabolized to lactate (anaerobic glycolysis). In anaerobic glycolysis, the key enzyme that converts pyruvate into lactate is encoded by the *Ldha* gene. The expression levels of *Ldha* in the outer zone of XpO placentas were significantly higher than in the other placentas (XpO vs. XX: *P* = 0.007; XpO vs. XY: *P* = 0.015; XpO vs. XmO: *P* < 0.001; Figure 2B, Supplementary Table 2), suggesting higher levels of anaerobic respiration specifically in the outer zone of the XpO placentas, where a higher incidence of GCs was found.

### The labyrinths of XO and wild-type placentas were comparable

Next, we determined the relative expression of TE markers [keratin 19 (*Krt19*), syncytin b (*Synb*), glial cells missing homolog 1 (*Gcm1*)] and endothelial markers [endoglin (*Eng*), platelet/endothelial cell adhesion molecule 1 (*Pecam1*)] in the labyrinth of the four types of placentas.

The expression levels of TE-marker *Krt19* in the labyrinth of XpO placenta were significantly lower than that in XX placenta (*P* = 0.024, Figure 2C, Supplementary Table 2). However, the TE-markers *Synb* and *Gcm1* were similarly expressed in the four types of placentas (Figure 2C, Supplementary Table 2). There was also no difference in the expression of endothelial-markers *Eng* and *Pecam1* in the labyrinth (Figure 2C, Supplementary Table 2). Together, the data suggests that the labyrinth of XO placentas may be similar to wild-type placentas.

**Figure 3:**
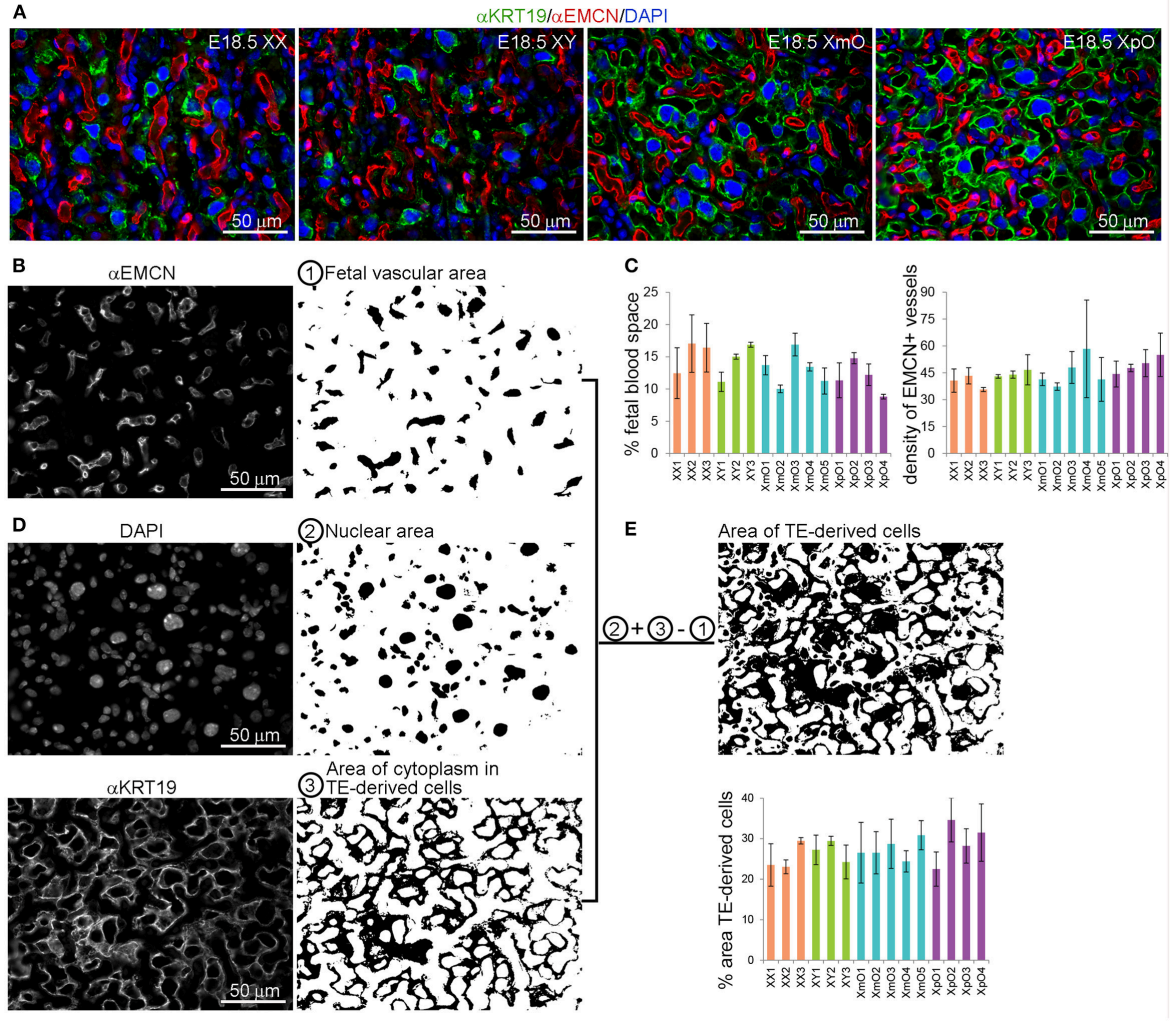
XO placentas show comparable fetal vascular area and TE-derived cell area in the labyrinth. **(A)** Representative medial placental sections from the different genotypes (XX, XY, XmO, XpO) immunostained for KRT19 and EMCN show the organization of the fetal vasculature and maternal blood space in the labyrinth. **(B)** To quantify the fetal vascular area and density, single channel images for EMCN-positive fetal capillaries are filled with black area ① and either the number of vessels counted (density of fetal vessels in that area) or the percentage of fetal vascular area is quantified (sum of the area of EMCN-positive blood vessels/total area of the image × 100). **(C)** Graph depicting the percentage (%) of area occupied by fetal vasculature and the density of EMCN-positive fetal blood vessels in the different genotypes analyzed (XX, XY, XmO, XpO). **(D)** To calculate the percentage of area occupied by TE-derived cells, the single channel images for DAPI ② and KRT19 ③ are converted to black-and-white, added digitally and finally from that area (②+③) the fetal capillary area is subtracted (②+③−①) (sum of the resulting black area/total area of the image × 100). **(E)** Graph depicts the percentage (%) of area occupied by TE-derived cells in the different genotypes analyzed (XX, XY, XmO, XpO).

To further confirm that, we quantified the area occupied by (EMCN-positive) fetal capillaries and (KRT19-positive) TE-derived cells in the labyrinth (*n* = 3–5 sections per individual placenta; *N* = 3 XX, *N* = 3 XY, *N* = 5 XmO, *N* = 4 XpO; Figure 3). For the quantification, single channel images of the labyrinth zone from medial placental sections immunostained for anti-EMCN (red) and anti-KRT19 (green), and DAPI (details shown in Figures 3B,D,E) were used. The area of the fetal vasculature (Figure 3B), considered the area occupied by the EMCN-positive fetal capillaries, was similar between XO and wild-type labyrinths (Figure 3C, left panel). Moreover, the vascular density, calculated as the number of fetal capillaries per image, was also comparable between XO and wild-type labyrinths (Figure 3C, right panel).

To quantify the area of occupied by TE-derived cells, the nuclei area (DAPI-positive) was merged with the KRT19-positive cytoplasmic staining of the TE derived cells. However, to exclude the nuclear area of the fetal vasculature, we subtracted the fetal vascular area (Figures 3B,D,E). In this way, we obtained the area occupied solely by TE-derived cells (cytoplasm and nuclei). We concluded that the area occupied by TE-derived cells in labyrinth was similar between XO and wild-type placentas (Figure 3E).

### At term, XpO placentas showed decreased *Xlr4b/4c* in the labyrinth zone

The X-linked *Xlr3b* has previously been identified as differentially expressed in XmO and XpO brains, but not in the placentas (Davies et al., 2005). In addition to *Xlr3b, Xlr4b/4c* were also reported to be differentially expressed between XmO and XpO brains, but at least *Xlr4c* not in placentas (Raefski and O'Neill, 2005). However, whole placentas were used for analysis and a possible regional regulation could have been missed. We therefore investigated the expression of *Xlr3b* and *Xlr4b/4c* separately in outer zone and labyrinth zone of term placenta.

**Figure 4:**
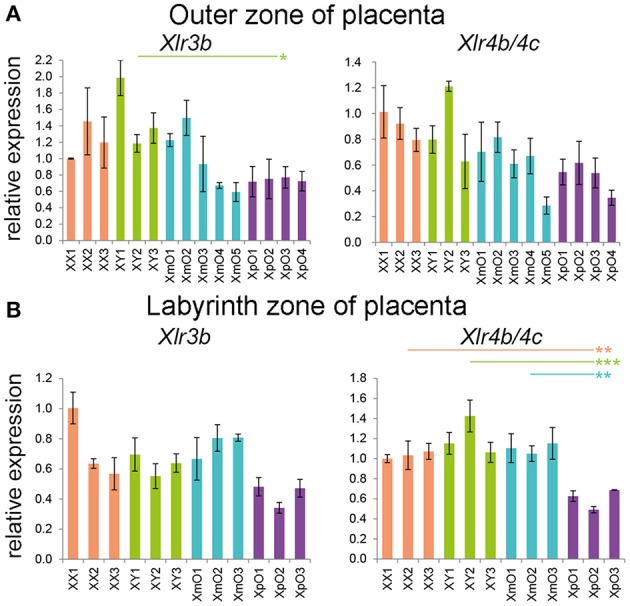
*Xlr* genes expression in the outer zone and labyrinth zone of term placentas. **(A,B)** Relative expression of *Xlr3b* and *Xlr4b/4c* in the outer zone **(A)** and labyrinth zone **(B)** of XX, XY, XmO, XpO placentas. Each bar represents mean ± standard deviation of technical triplicates of a single placenta. Significant *P*-values between genotypes are indicated by ^*^P < 0.05, ^**^P < 0.01, and ^***^P < 0.001.

We did not observe significantly differential expression of *Xlr3b* and *Xlr4b/4c* in the XpO and XmO outer zones (Figure 4A, Supplementary Table 2), even though XpO outer zones had significantly lower *Xlr3b* than XY outer zones (*P* = 0.030; Figure 4A, Supplementary Table 2). In the labyrinth zone, the expression levels of *Xlr3b* were comparable between genotypes (Figure 4B, Supplementary Table 2), but surprisingly XpO labyrinth zone had significantly lower expression levels of *Xlr4b/4c* than the other placentas (XpO vs. XX: *P* = 0.006; XpO vs. XY: *P* < 0.001; XpO vs. XmO: *P* = 0.003; Figure 4B, Supplementary Table 2).

### At term, XmO placentas contained higher incidence of fibrin nodules in the maternal arterial sinuses adjacent to the chorionic plate

**Figure 5:**
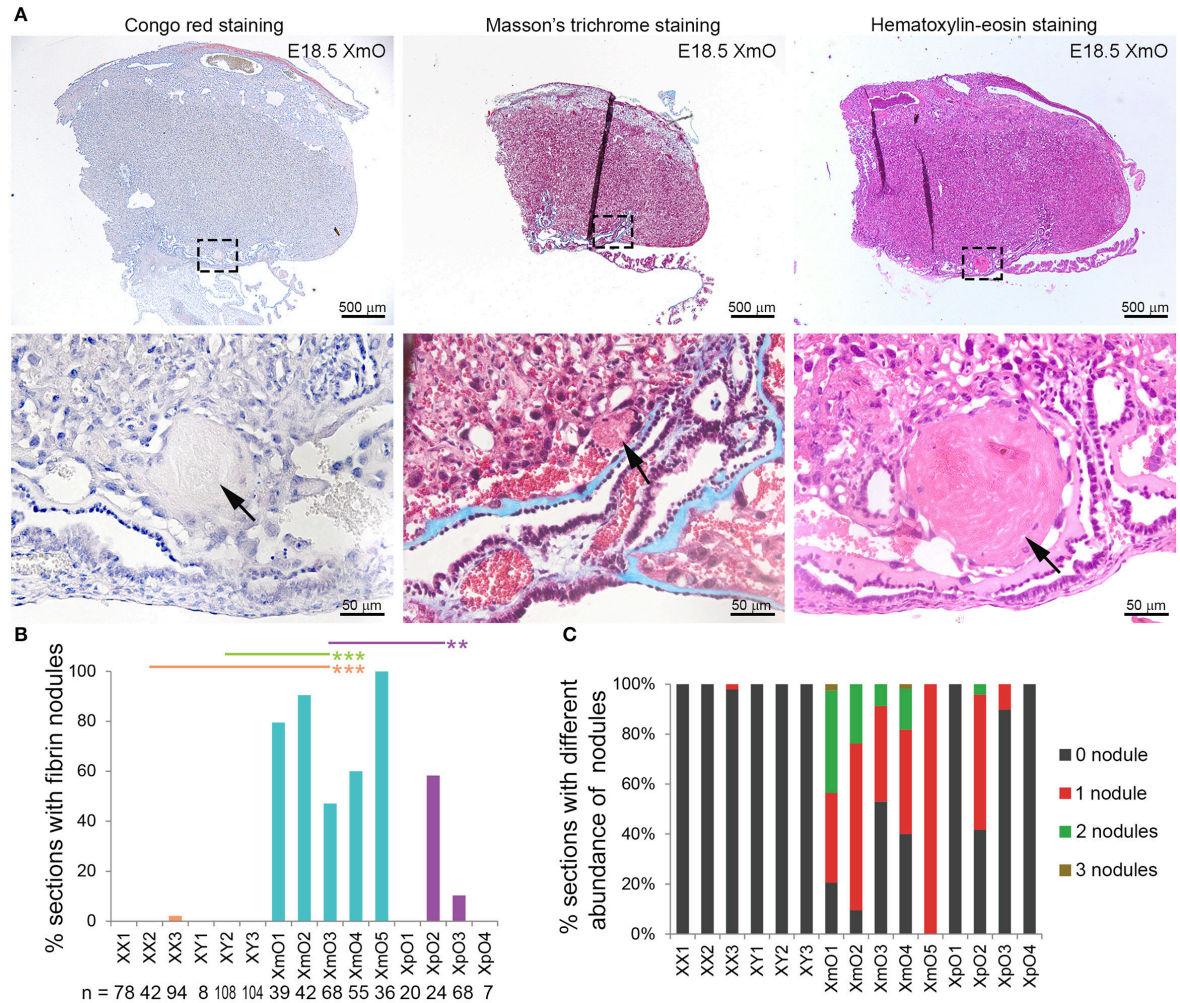
Identifications and quantification of fibrin deposits in term placentas. **(A)** Representative medial XmO placenta sections with Congo red, Masson's trichrome and Hematoxylin-Eosin staining. The dashed boxes in top panels are shown as magnification in bottom panels. Black arrows point to fibrin deposits. **(B)** Graph depicting the percentage (%) of placental sections with fibrin nodules per histological section (*n*) in different genotypes. Significant *P*-values between XmO placentas and the other genotypes are indicated by ^**^P < 0.01 and ^***^P < 0.001. **(C)** Graph depicting the percentage (%) of placental sections with different abundance of fibrin nodules per histological section (n in **B**) in different genotypes.

Interestingly, the XmO placentas contained small nodules, partially occluding some maternal arterial sinuses proximal of the chorionic plate (black arrow in Figure 1A). After Congo red, Masson's trichrome and Hematoxylin-eosin staining, we concluded that those were fibrin deposits (Figure 5A).

To quantify the incidence of fibrin nodules in the different placentas (*N* = 3 XX, *N* = 3 XY, *N* = 5 XmO, *N* = 4 XpO), we manually counted the number of fibrin nodules present in medial placental sections. All XmO placentas contained fibrin deposits in most sections analyzed, whereas the nodules were not observed in most of the other placentas (XmO vs. XX: *P* = 0.0007; XmO vs. XY: *P* = 0.0007; XmO vs. XpO: *P* = 0.0019; Figure 5B). On average, per section one or two nodules were observed (Figures 5A,C).

## Discussion

The lack of a second sex chromosome in XO mouse embryos leads to the development of smaller trophectoderm ectoplacental cones and, in some cases, pregnancy loss (Burgoyne et al., 1983b; Hunt, 1991; Thornhill and Burgoyne, 1993; Jamieson et al., 1998; Ishikawa et al., 2003). Our study on XO placental abnormalities in mice shows that inheriting the single X chromosome from the paternal or maternal side leads to different placental phenotypes: E18.5 XpO placentas contained a larger area of GCs in the outer zone with a possible consequent increase in anaerobic glycolysis and/or oxygen availability. This does not seem to be the case in XmO placentas. By contrast, XmO placentas show increased incidence of small fibrin nodules in maternal arterial sinuses proximal of the chorionic plate.

The placenta is sensitive to defects in epigenetic regulation, such as regulation of imprinted genes and imprinted X inactivation (Hemberger, 2002; Lefebvre, 2012; Himes et al., 2013). As such, it is not surprising that many imprinted and X-linked genes are expressed in the placenta and regulate metabolism and growth (Hemberger, 2002; Lefebvre, 2012; Sandovici et al., 2012). The higher incidence of GCs in the outer zone of XpO placentas may be such an adaptive response to placental insufficiency. Studies in mice show that XpO fetuses have a higher risk of being lost during pregnancy, whereas XmO fetuses generally have a better chance of surviving to term (Hunt, 1991; Jamieson et al., 1998). In XX fetuses, the Xp is preferentially inactivated in the TE-derived tissues (Harper et al., 1982). Thus, in both females and males, it is the Xm that is active in TE-derived tissues during placental development. Epigenetic differences between Xp and Xm, including genome-wide differences in chromatin condensation or the existence of X-linked paternally imprinted genes in the placenta, could explain why XpO embryos have a higher risk of being lost during pregnancy.

Alternatively, as a smaller trophectoderm ectoplacental cone in XO embryos is often associated with a delay in embryonic development (Thornhill and Burgoyne, 1993; Jamieson et al., 1998; Ishikawa et al., 2003), the alteration observed in GCs in outer zone of XpO placentas could reflect a general small delay in development instead of an (active) adaptive response. Interestingly, it has been shown that both the number of GCs and the volume of the junctional zone increases during gestation, peaks at E16.5, followed by reduction until birth (Coan et al., 2004, 2006). Therefore, if the physiological regression of GCs in XpO placentas was delayed, this would result in relatively more GCs and increased size of the outer zone at E18.5 compared to wild-type placentas.

Abnormal GC numbers, related to impaired glucose transport and glycogen metabolism in placenta (Redline et al., 1993; Sibley et al., 2004), have been reported in several mutant mouse placentas, including that of X-linked and imprinted genes, and usually result in runting. Opposite phenotypes regarding GCs numbers can still develop in mouse mutants showing a normal placenta at E10.5. As the XpO phenotype, maternally inherited defects in X-linked *Evx1* also showed higher numbers of GCs, spongiotrophoblasts and secondary TGCs by E14.5 and the mutant pups were smaller at birth (Li and Behringer, 1998). By contrast, *Cited2 KO* and the imprinted (paternally-expressed) *Igf2* KO mice show severe reduction of numbers of GCs, spongiotrophoblasts and secondary TGCs at E12.5–13.5 (Lopez et al., 1996; Withington et al., 2006), but this also resulted in reduced weight at birth (Sibley et al., 2004); whereas the imprinted (maternally-expressed) *Cdkn1c* (or *p57*^*Kip*2^) KO showed placentomegaly, with larger labyrinth zone and excess of spongiotrophoblasts, but normal number of GCs and TGCs and no difference in the weight of embryos (Takahashi et al., 2000).

The detected significantly higher expression of *Ldha* in outer zone of XpO placentas indicates a switch from aerobic to anaerobic glycolysis in outer zone of XpO placentas. *Ldha* expression is reported to be increased in human primary placental trophoblast cells under hypoxic conditions (Kay et al., 2007). Interestingly, transcriptomics analysis between XX and XO human fibroblasts revealed differences in glucose metabolism (Rajpathak et al., 2014), but the parental origin of the X chromosome was unclear. Moreover, women with Turner syndrome are characterized by increased size of type IIa muscle fibers in addition to impaired glucose tolerance and insulin resistance, indicating diminished oxygen and substrate supply for metabolic processes (Gravholt et al., 2001). It is also reported that Turner syndrome women have increased anaerobic glycolysis and lactic acid production during exercise, compared to a control group (Wells et al., 2013).

It is unclear whether Xlr factors are directly involved in glucose metabolism, but they could impact on cell differentiation in the outer zone toward GC cells. We observed similar expression of *Xlr3b* and *Xlr4b/4c* in XpO and XmO outer zones suggesting that these genes are not involved in the production of GCs and are not imprinted in the (TE-derived) outer zone. However, the biological significance of the specific decrease in *Xlr4b/4c* in XpO labyrinth remains to be investigated. If this decrease is not due to imprinting in TE-derived labyrinth cells (mononuclear trophoblast and syncytiotrophoblast cells), then perhaps *Xlr4b/c* could be imprinted and silenced on the Xp in the EPI-derived endothelial cells of the labyrinth, in line with the reported imprint in the brain (Raefski and O'Neill, 2005). This could explain the 40% reduction in expression observed.

Small fibrin nodules, most probably from maternal blood-clots, were observed in the maternal sinus of all XmO placentas, but not in most of the other placentas. Interestingly, TE-derived cells at the fetal-maternal interface in both mouse and human exhibit endothelial-like properties (endovascular extravillous trophoblast cells and syncytiotrophoblast cells in humans; syncytiotrophoblast cells in mice) and seem to be involved in the regulation of coagulation during pregnancy (Sood et al., 2006). Fibrin deposits are occasionally described in the labyrinth and spongiotrophoblast area of mouse placentas (Vogt et al., 1996; Redecha et al., 2009); and in the perivillous space, associated with local syncytial denudation, in human placentas (Nelson et al., 1990; Khan et al., 2011). Excessive fibrin deposits at the fetal-maternal interface early during development, such as in *Procr* (or *Epcr*) KO embryos results in severe placental thrombosis and lethality at E10.5 (Gu et al., 2002); whereas in *Wnt2* KO embryos showed fibrin deposits between E14 and E18 with maternal blood accumulation in the labyrinth zone, resulting in 50% viability and smaller pups at birth (Monkley et al., 1996). The fibrin deposits in XmO placentas indicate excessive activation of the coagulation cascade in the maternal circulation, but this obstruction was not as severe as in *Wnt2* KO mice and, as such, does not seem to be pathological.

In conclusion, mouse embryos with a single Xp have a lower chance than XmO embryos to survive to term due to placental insufficiency. Here, we show that XmO and XpO term placentas differ significantly in the amount of GCs in the outer zone and that XpO placentas may have shifted toward anaerobic glycolysis. This shift in glucose metabolism does not seem to be a direct consequence of altered expression of X-linked genes involved in this metabolism (although *Pgk1* expression differs between XmO and XpO outer zones), but rather a consequence of an altered cellular composition of the XpO outer zone (large GCs area) due to placental adaptive response earlier during development. Our findings highlight the need to investigate glucose metabolism in the placenta of human Turner patients, which may provide individual potential therapeutic strategies for Turner Syndrome.

## Authors contributions

NH, SL, JC, DS, LP, SC designed the study. NH, SL, JC, IN, MB, DS, LV, LP, SC performed experiments and/or analyzed data. NH, SL, SC wrote the manuscript. All authors contributed critical comments and corrections and gave approval for publication.

### Conflict of interest statement

The authors declare that the research was conducted in the absence of any commercial or financial relationships that could be construed as a potential conflict of interest.

## References

[B1] Adelman D. M. Gertsenstein M. Nagy A. Simon M. C. Maltepe E. (2000). Placental cell fates are regulated *in vivo* by HIF-mediated hypoxia responses. Genes Dev. 14, 3191–3203. 10.1101/gad.853700 11124810 PMC317149

[B2] Álvarez-Nava F. Lanes R. Quintero J. M. Miras M. Fideleff H. Mericq V.. (2013). Effect of the parental origin of the X-chromosome on the clinical features, associated complications, the two-year-response to growth hormone (rhGH) and the biochemical profile in patients with turner syndrome. Int. J. Pediatr. Endocrinol. 2013:10. 10.1186/1687-9856-2013-10 23731950 PMC3679778

[B3] Burgoyne P. S. Evans E. P. Holland K. (1983a). XO monosomy is associated with reduced birthweight and lowered weight gain in the mouse. J. Reprod. Fertil. 68, 381–385. 10.1530/jrf.0.0680381 6864653

[B4] Burgoyne P. S. Tam P. P. Evans E. P. (1983b). Retarded development of XO conceptuses during early pregnancy in the mouse. J. Reprod. Fertil. 68, 387–393. 10.1530/jrf.0.0680387 6864654

[B5] Coan P. M. Conroy N. Burton G. J. Ferguson-Smith A. C. (2006). Origin and characteristics of glycogen cells in the developing murine placenta. Dev. Dyn. 235, 3280–3294. 10.1002/dvdy.20981 17039549

[B6] Coan P. M. Ferguson-Smith A. C. Burton G. J. (2004). Developmental dynamics of the definitive mouse placenta assessed by stereology. Biol. Reprod. 70, 1806–1813. 10.1095/biolreprod.103.024166 14973263

[B7] Cockwell A. MacKenzie M. Youings S. Jacobs P. (1991). A cytogenetic and molecular study of a series of 45,X fetuses and their parents. J. Med. Genet. 28, 151–155. 10.1136/jmg.28.3.151 1675683 PMC1016795

[B8] Davies W. Isles A. Smith R. Karunadasa D. Burrmann D. Humby T.. (2005). Xlr3b is a new imprinted candidate for X-linked parent-of-origin effects on cognitive function in mice. Nat. Genet. 37, 625–629. 10.1038/ng1577 15908950

[B9] de Mello J. C. M. de Araujo E. S. S. Stabellini R. Fraga A. M. de Souza J. E. S. Sumita D. R.. (2010). Random X inactivation and extensive mosaicism in human placenta revealed by analysis of allele-specific gene expression along the X chromosome. PLoS ONE 5:e10947. 10.1371/journal.pone.0010947 20532033 PMC2881032

[B10] de Melo Bernardo A. Heeren A. M. van Iperen L. Fernandes M. G. He N. Anjie S.. (2015). Meiotic wave adds extra asymmetry to the development of female chicken gonads. Mol. Reprod. Dev. 82, 774–786. 10.1002/mrd.22516 26096940 PMC5034815

[B11] Deng X. Berletch J. B. Nguyen D. K. Disteche C. M. (2014). X chromosome regulation: diverse patterns in development, tissues and disease. Nat. Rev. Genet. 15, 367–378. 10.1038/nrg3687 24733023 PMC4117651

[B12] Dimasuay K. G. Boeuf P. Powell T. L. Jansson T. (2016). Placental responses to changes in the maternal environment determine fetal growth. Front. Physiol. 7:12. 10.3389/fphys.2016.00012 26858656 PMC4731498

[B13] Fowden A. L. Sferruzzi-Perri A. N. Coan P. M. Constancia M. Burton G. J. (2009). Placental efficiency and adaptation: endocrine regulation. J. Physiol. 587(Pt 14), 3459–3472. 10.1113/jphysiol.2009.173013 19451204 PMC2742275

[B14] Ganguly A. Collis L. Devaskar S. U. (2012). Placental glucose and amino acid transport in calorie-restricted wild-type and Glut3 null heterozygous mice. Endocrinology 153, 3995–4007. 10.1210/en.2011-1973 22700768 PMC3404359

[B15] Gravholt C. H. Nyholm B. Saltin B. Schmitz O. Christiansen J. S. (2001). Muscle fiber composition and capillary density in Turner syndrome: evidence of increased muscle fiber size related to insulin resistance. Diabetes Care 24, 1668–1673. 10.2337/diacare.24.9.1668 11522717

[B16] Gu J.-M. Crawley J. T. Ferrell G. Zhang F. Li W. Esmon N. L.. (2002). Disruption of the endothelial cell protein C receptor gene in mice causes placental thrombosis and early embryonic lethality. J. Biol. Chem. 277, 43335–43343. 10.1074/jbc.M207538200 12218060

[B17] Hamada H. Okae H. Toh H. Chiba H. Hiura H. Shirane K.. (2016). Allele-specific methylome and transcriptome analysis reveals widespread imprinting in the human placenta. Am. J. Hum. Genet. 99, 1045–1058. 10.1016/j.ajhg.2016.08.021 27843122 PMC5097938

[B18] Harper M. I. Fosten M. Monk M. (1982). Preferential paternal X inactivation in extraembryonic tissues of early mouse embryos. J. Embryol. Exp. Morphol. 67, 127–135. 7086330

[B19] Heeren A. M. van Iperen L. Klootwijk D. B. de Melo Bernardo A. Roost M. S. Gomes Fernandes M. M.. (2015). Development of the follicular basement membrane during human gametogenesis and early folliculogenesis. BMC Dev. Biol. 15:4. 10.1186/s12861-015-0054-0 25605128 PMC4307144

[B20] Hemberger M. (2002). The role of the X chromosome in mammalian extra embryonic development. Cytogenet. Genome Res. 99, 210–217. 10.1159/000071595 12900566

[B21] Higgins J. S. Vaughan O. R. Fernandez de Liger E. Fowden A. L. Sferruzzi-Perri A. N. (2016). Placental phenotype and resource allocation to fetal growth are modified by the timing and degree of hypoxia during mouse pregnancy. J. Physiol. 594, 1341–1356. 10.1113/JP271057 26377136 PMC4771776

[B22] Himes K. P. Koppes E. Chaillet J. R. (2013). Generalized disruption of inherited genomic imprints leads to wide-ranging placental defects and dysregulated fetal growth. Dev. Biol. 373, 72–82. 10.1016/j.ydbio.2012.10.010 23085235 PMC3508140

[B23] Hunt P. A. (1991). Survival of XO mouse fetuses: effect of parental origin of the X chromosome or uterine environment? Development 111, 1137–1141. 1879355 10.1242/dev.111.4.1137

[B24] Ishikawa H. Rattigan A. Fundele R. Burgoyne P. S. (2003). Effects of sex chromosome dosage on placental size in mice. Biol. Reprod. 69, 483–488. 10.1095/biolreprod.102.012641 12700203

[B25] Jacobs P. Hassold T. Harvey J. May K. (1989). The origin of sex chromosome aneuploidy. Prog. Clin. Biol. Res. 311, 135. 2528150

[B26] Jamieson R. V. Tan S. S. Tam P. P. (1998). Retarded postimplantation development of X0 mouse embryos: impact of the parental origin of the monosomic X chromosome. Dev. Biol. 201, 13–25. 10.1006/dbio.1998.8972 9733570

[B27] Jansson T. (2016). Placenta plays a critical role in maternal-fetal resource allocation. Proc. Natl. Acad. Sci. U.S.A. 113, 11066–11068. 10.1073/pnas.1613437113 27660237 PMC5056066

[B28] Kay H. H. Zhu S. Tsoi S. (2007). Hypoxia and lactate production in trophoblast cells. Placenta 28, 854–860. 10.1016/j.placenta.2006.11.011 17275903

[B29] Khan H. M. Khan M. Y. Minhas L. A. (2011). Histological study of the developing mouse placenta. J. Rawalpindi Med. Coll. 15, 116–119.

[B30] Ko J. M. Kim J. M. Kim G. H. Lee B. H. Yoo H. W. (2010). Influence of parental origin of the X chromosome on physical phenotypes and GH responsiveness of patients with Turner syndrome. Clin. Endocrinol. 73, 66–71. 10.1111/j.1365-2265.2010.03782.x 20148908

[B31] Lefebvre L. (2012). The placental imprintome and imprinted gene function in the trophoblast glycogen cell lineage. Reprod. Biomed. Online 25, 44–57. 10.1016/j.rbmo.2012.03.019 22560119 PMC3819294

[B32] Li Y. Behringer R. R. (1998). Esx1 is an X-chromosome-imprinted regulator of placental development and fetal growth. Nat. Genet. 20, 309–311. 10.1038/3129 9806555

[B33] Lopez M. Dikkes P. Zurakowski D. Villa-Komaroff L. (1996). Insulin-like growth factor II affects the appearance and glycogen content of glycogen cells in the murine placenta. Endocrinology 137, 2100–2108. 10.1210/endo.137.5.8612553 8612553

[B34] Mathur A. Stekol L. Schatz D. MacLaren N. Scott M. Lippe B. (1991). The parental origin of the single X chromosome in Turner syndrome: lack of correlation with parental age or clinical phenotype. Am. J. Hum. Genet. 48, 682. 1673045 PMC1682964

[B35] Monkley S. J. Delaney S. J. Pennisi D. J. Christiansen J. H. Wainwright B. J. (1996). Targeted disruption of the Wnt2 gene results in placentation defects. Development 122, 3343–3353. 8951051 10.1242/dev.122.11.3343

[B36] Monroy N. López M. Cervantes A. García-Cruz D. Zafra G. Canún S.. (2002). Microsatellite analysis in Turner syndrome: parental origin of X chromosomes and possible mechanism of formation of abnormal chromosomes. Am. J. Med. Genet. 107, 181–189. 10.1002/ajmg.10113 11807897

[B37] Nelson D. M. Crouch E. C. Curran E. M. Farmer D. R. (1990). Trophoblast interaction with fibrin matrix. Epithelialization of perivillous fibrin deposits as a mechanism for villous repair in the human placenta. Am. J. Pathol. 136, 855–865. 2327472 PMC1877640

[B38] Okamoto I. Patrat C. Thepot D. Peynot N. Fauque P. Daniel N.. (2011). Eutherian mammals use diverse strategies to initiate X-chromosome inactivation during development. Nature 472, 370–374. 10.1038/nature09872 21471966

[B39] Payer B. Lee J. T. (2008). X chromosome dosage compensation: how mammals keep the balance. Annu. Rev. Genet. 42, 733–772. 10.1146/annurev.genet.42.110807.091711 18729722

[B40] Payer B. Lee J. T. (2014). Coupling of X-chromosome reactivation with the pluripotent stem cell state. RNA Biol. 11, 798–807. 10.4161/rna.29779 25137047 PMC4179954

[B41] Penaherrera M. Jiang R. Avila L. Yuen R. Brown C. Robinson W. (2012). Patterns of placental development evaluated by X chromosome inactivation profiling provide a basis to evaluate the origin of epigenetic variation. Hum. Reprod. 27, 1745–1753. 10.1093/humrep/des072 22431562 PMC3357192

[B42] Petropoulos S. Panula S. P. Schell J. P. Lanner F. (2016). Single-cell RNA sequencing: revealing human pre-implantation development, pluripotency and germline development. J. Intern. Med. 280, 252–264. 10.1111/joim.12493 27046137

[B43] Raefski A. S. O'Neill M. J. (2005). Identification of a cluster of X-linked imprinted genes in mice. Nat. Genet. 37, 620–624. 10.1038/ng1567 15908953

[B44] Rajpathak S. N. Vellarikkal S. K. Patowary A. Scaria V. Sivasubbu S. Deobagkar D. D. (2014). Human 45, X fibroblast transcriptome reveals distinct differentially expressed genes including long noncoding RNAs potentially associated with the pathophysiology of Turner syndrome. PLoS ONE 9:e100076. 10.1371/journal.pone.0100076 24932682 PMC4059722

[B45] Ranke M. B. Saenger P. (2001). Turner's syndrome. Lancet 358, 309–314. 10.1016/S0140-6736(01)05487-3 11498234

[B46] Redecha P. van Rooijen N. Torry D. Girardi G. (2009). Pravastatin prevents miscarriages in mice: role of tissue factor in placental and fetal injury. Blood 113, 4101–4109. 10.1182/blood-2008-12-194258 19234141 PMC2943752

[B47] Redline R. W. Chernicky C. L. Tan H. Q. Ilan J. Ilan J. (1993). Differential expression of insulin-like growth factor-II in specific regions of the late (post day 9.5) murine placenta. Mol. Reprod. Dev. 36, 121–129. 10.1002/mrd.1080360202 8257562

[B48] Romijn H. J. van Uum J. F. Emmering J. Goncharuk V. Buijs R. M. (1999). Colocalization of VIP with AVP in neurons of the human paraventricular, supraoptic and suprachiasmatic nucleus. Brain Res. 832, 47–53. 10.1016/S0006-8993(99)01468-7 10375651

[B49] Saenger P. (1996). Turner's syndrome. N. Engl. J. Med. 335, 1749–1754. 10.1056/NEJM199612053352307 8929268

[B50] Sagi L. Zuckerman-Levin N. Gawlik A. Ghizzoni L. Buyukgebiz A. Rakover Y.. (2007). Clinical significance of the parental origin of the X chromosome in turner syndrome. J. Clin. Endocrinol. Metab. 92, 846–852. 10.1210/jc.2006-0158 17192299

[B51] Sandovici I. Hoelle K. Angiolini E. Constancia M. (2012). Placental adaptations to the maternal-fetal environment: implications for fetal growth and developmental programming. Reprod. Biomed. Online 25, 68–89. 10.1016/j.rbmo.2012.03.017 22560117

[B52] Semenza G. L. Roth P. H. Fang H. M. Wang G. L. (1994). Transcriptional regulation of genes encoding glycolytic enzymes by hypoxia-inducible factor 1. J. Biol. Chem. 269, 23757–23763. 8089148

[B53] Sibley C. Coan P. Ferguson-Smith A. Dean W. Hughes J. Smith P.. (2004). Placental-specific insulin-like growth factor 2 (Igf2) regulates the diffusional exchange characteristics of the mouse placenta. Proc. Natl. Acad. Sci. U.S.A. 101, 8204–8208. 10.1073/pnas.0402508101 15150410 PMC419581

[B54] Silva J. Nichols J. Theunissen T. W. Guo G. van Oosten A. L. Barrandon O.. (2009). Nanog is the gateway to the pluripotent ground state. Cell 138, 722–737. 10.1016/j.cell.2009.07.039 19703398 PMC3437554

[B55] Solano M. E. Thiele K. Kowal M. K. Arck P. C. (2016). Identification of suitable reference genes in the mouse placenta. Placenta 39, 7–15. 10.1016/j.placenta.2015.12.017 26992668

[B56] Sood R. Kalloway S. Mast A. E. Hillard C. J. Weiler H. (2006). Fetomaternal cross talk in the placental vascular bed: control of coagulation by trophoblast cells. Blood 107, 3173–3180. 10.1182/blood-2005-10-4111 16380449 PMC1895751

[B57] Takahashi K. Kobayashi T. Kanayama N. (2000). p57Kip2 regulates the proper development of labyrinthine and spongiotrophoblasts. Mol. Hum. Reprod. 6, 1019–1025. 10.1093/molehr/6.11.1019 11044465

[B58] Thornhill A. R. Burgoyne P. S. (1993). A paternally imprinted X chromosome retards the development of the early mouse embryo. Development 118, 171–174. 8375333 10.1242/dev.118.1.171

[B59] Tuttle S. Stamato T. Perez M. L. Biaglow J. (2000). Glucose-6-phosphate dehydrogenase and the oxidative pentose phosphate cycle protect cells against apoptosis induced by low doses of ionizing radiation. Radiat. Res. 153, 781–787. 10.1667/0033-7587(2000)153[0781:GPDATO]2.0.CO;2 10825753

[B60] Uematsu A. Yorifuji T. Muroi J. Kawai M. Mamada M. Kaji M.. (2002). Parental origin of normal X chromosomes in Turner syndrome patients with various karyotypes: implications for the mechanism leading to generation of a 45, X karyotype. Am. J. Med. Genet. 111, 134–139. 10.1002/ajmg.10506 12210339

[B61] Vogt E. Ng A.-K. Rote N. S. (1996). A model for the antiphospholipid antibody syndrome: monoclonal antiphosphatidylserine antibody induces intrauterine growth restriction in mice. Am. J. Obstet. Gynecol. 174, 700–707. 10.1016/S0002-9378(96)70453-2 8623810

[B62] Wegrzyn P. Faro C. Falcon O. Peralta C. Nicolaides K. (2005). Placental volume measured by three-dimensional ultrasound at 11 to 13+ 6 weeks of gestation: relation to chromosomal defects. Ultrasound obstet. Gynecol. 26, 28–32. 10.1002/uog.1923 15937964

[B63] Wells G. D. O'Gorman C. S. Rayner T. Caterini J. Thompson S. Bradley T.. (2013). Skeletal muscle abnormalities in girls and adolescents with Turner syndrome. J. Clin. Endocrinol. Metab. 98, 2521–2527. 10.1210/jc.2012-4016 23553856

[B64] Withington S. Scott A. Saunders D. Floro K. L. Preis J. Michalicek J.. (2006). Loss of Cited2 affects trophoblast formation and vascularization of the mouse placenta. Dev. Biol. 294, 67–82. 10.1016/j.ydbio.2006.02.025 16579983

[B65] Zechner U. Reule M. Burgoyne P. S. Schubert A. Orth A. Hameister H.. (1997). Paternal transmission of X-linked placental dysplasia in mouse interspecific hybrids. Genetics 146, 1399–1405. 9258682 10.1093/genetics/146.4.1399 PMC1208083

[B66] Zhao F. Q. Keating A. F. (2007). Functional properties and genomics of glucose transporters. Curr. Genomics 8, 113–128. 10.2174/138920207780368187 18660845 PMC2435356

